# Hypogonadism among patients with transfusion-dependent thalassemia: a cross-sectional study

**DOI:** 10.1097/MS9.0000000000000947

**Published:** 2023-06-08

**Authors:** Taha O. Mahwi, Zagros G. Rashid, Shaho F. Ahmed

**Affiliations:** a Internal Medicine Department, College of Medicine, University of; b Shar Hospital, Ministry of Health, Sulaimaniyah City; c Smart Health Tower, Madam Mitterrand Street, Sulaimani, Kurdistan, Iraq

**Keywords:** chelating agents, hypogonadism, iron overload, thalassemia

## Abstract

Patients with thalassemia major are liable to have complications from blood transfusion and iron excess despite using iron-chelating agents. It is common to have endocrine complications in these patients. Hypogonadism is one of the most common complications in thalassemic patients. Early detection and treatment are essential to restore puberty and prevent complications from hypogonadism.

**Patients and methods:**

The authors conducted this cross-sectional study in the Kurdistan Region of Iraq, from 1 July 2022, to 1 December 2022. Eighty patients with β-thalassemia major who were referred to the endocrinology clinic were enrolled. The patients were evaluated sequentially, starting with a history, followed by a clinical examination and laboratory tests related to endocrine disease. Those who met the inclusion criteria were enrolled in the study, while the others were excluded.

**Results:**

Out of 80 patients with major thalassemia who were referred to the endocrinology clinic, 53 (66.3%) were female, and 27 (33.7%) were male, with a mean (SD) age of 24.86±7.66 (14–59) years. Fifty-five (68.75%) of them had hypogonadism, three patients (3.8%) had hypothyroidism, while two (2.5%) had hypoparathyroidism. Five patients (6.3%) had diabetes. None of the patients had adrenal insufficiency. The mean ferritin level in thalassemic patients with hypogonadism and thalassemic patients without hypogonadism was 2326±2625 ng/ml vs. 1220±2625 ng/ml.

**Conclusion:**

To minimize the risk of endocrinopathy in patients with thalassemia major, they should receive blood transfusions regularly and initiate chelating agents early, because the leading cause of endocrinopathy in thalassemic patients is related to the severity of anemia and iron overload.

## Introduction

Highlights

- Patients with thalassemia major are liable to have complications from blood transfusion and iron excess despite using iron-chelating agents.
- Hypogonadism is one of the most common complications in thalassemic patients.
- The leading cause of endocrinopathy in thalassemic patients is related to the severity of anemia and iron overload.
- This study aims to evaluate the prevalence of hypogonadism in referred patients with thalassemia major, assess the type of hypogonadism.

β-thalassemia is an autosomal recessive disorder that occurs as a result of either a lack of synthesis of the β-globin chains of hemoglobin (β 0) or reduced synthesis of that chain (β 1). It is regarded as one of the most common diseases inherited as a single gene disorder, and about 270 million carriers are present globally^1,2^. Three types of β-thalassemia are described: thalassemia major, which is the most severe form that needs regular blood transfusions, and thalassemia intermedia and minor, which are the mild forms of the disease^3^.

Regular blood transfusion is associated with the accumulation of iron in different tissues in the body^4^. The commonly reported organs that are liable for marked iron deposition are the liver, pancreas, pituitary, thyroid, parathyroid, heart, adrenal, gonads, renal medulla, spleen, and bone marrow^5^. Although the primary cause of death in these patients is heart disease, endocrinopathy, especially hypogonadism, remains the most common manifestation of β-thalassemia major^6^.

Puberty is defined as the development of secondary sexual characteristics, the initiation of fertility, the achievement of adult height, and psychosocial well-being^7^. The hypothalamic-pituitary-gonadal axis remains inactive until puberty, after which it will start to maintain growth and initiate secondary sexual characteristics. Delayed puberty is a failure to achieve secondary sexual characteristics by the age of 14 in males and 13 in females, which may be caused by congenital or acquired diseases and has significant effects on both physical and psychosocial well-being^8^.

Hypogonadism manifests in males as a testicular volume of less than 4 ml, while in females, it is marked by the absence of breast development by age 16^9,10^. Hypogonadism in patients with thalassemia major is caused by iron deposition in the pituitary gland, gonads, or both^11^. It has been shown that gonadotrophs need a higher iron level, are more sensitive than other anterior pituitary cells, and are most affected by iron overload^12^. The only effective way to get rid of iron overload is the use of chelating agents^13^. A study conducted in Italy showed a lack of benefit in using iron-chelating agents to prevent the endocrine complications of thalassemia major^14^. In contrast, other studies suggested that using iron-chelating agents reduces the risk of complications, especially endocrinopathy from transfusion-dependent thalassemia, and is associated with a better quality of life^15,16^. The higher the level of ferritin, the more the risk of complications from thalassemia^17^.

This study aims to evaluate the prevalence of hypogonadism in referred patients with thalassemia major, assess the type of hypogonadism, and study the relationship with precipitating factors and other endocrinopathies.

## Patients and methods

### Study protocol

This cross-sectional study was conducted in accordance with Strengthening the Reporting of Cohort Studies in Surgery (STROCSS)^18^.

The present study enrolled 80 patients with β-thalassemia major referred to the endocrinology clinic in the Kurdistan Region of Iraq.

The referred patients were evaluated first by taking a detailed clinical history that included the patients’ demographics, referral purpose, past medical and surgical history, use of drugs (especially chelating agents and compliance with this kind of therapy), the interval between blood transfusion sessions, and a focused review of symptoms related to the reproductive system. This was followed by a clinical examination, including Tanner staging, height, weight, and blood pressure measurement. Tanner staging, which includes five stages and is different for males and females^19,^ was used to decide clinically whether the patient had achieved puberty and whether they were hypogonadal. These findings were then corroborated with relevant laboratory investigations.

### Investigations

Basic biochemical and hormonal assays performed included a complete blood count, alanine transaminase, aspartate aminotransferase, urea, creatinine, thyroid stimulating hormone, triiodothyronine, thyroxine, serum calcium, serum phosphorus, vitamin D3, parathyroid hormone, luteinizing hormone (LH), follicle-stimulating hormone (FSH), estradiol in females, and testosterone in males.

A DEXA scan (T score:>−1 regarded as normal, −1 to −2.5 as osteopenia, and<−2.5 as osteoporotic)^20^ was performed for all patients.

### Inclusion criteria

All patients with thalassemia major referred to the endocrinology department were included in the study.

### Exclusion criteria

Patients were excluded from the study if they were younger than 14 years old or had a history suggesting other causes of hypogonadism rather than thalassemia (for example, a history of trauma to the gonads or the head, radiation, drugs known to cause hypogonadism) and those who had undergone bone marrow transplantation.

### Data collection and analysis

Data were taken from the patient’s record and from the questionnaire. Data collection, entry, and coding were done using Microsoft Excel Version 2019. The results are presented as the mean±SD. Qualitative data were presented as frequencies and percentages. Data analysis was performed using Statistical Package for the Social Sciences (SPSS) Version 26. Continuous variables were compared by the Student’s *t*-test, and discrete variables were analyzed using the *χ* ^2^-test.

## Results

In this cross-sectional study, 80 patients with β-thalassemia major referred to the endocrinology clinic were enrolled (Table 1).

**Table 1:** Baseline characteristics.

Variables	No. Cases/ frequency
Demographics	
Age in years (mean±SD)	24.86±7.66
Age range (min, max)	(14–59)
Sex	
Male	27 (33.7%)
Female	53 (66.3%)
Spleen	
Splenectomy	49 (61.3%)
Normal	21 (38.7%)
Chelating agent use	
Properly	46 (57.5%)
Improperly	34 (42.5%)
Interval between two sessions of blood transfusion (mean±SD)	20.83±5.61
Interval between two sessions of blood transfusion (min, max)	(14–35) days
Comorbidity	
Diabetes mellitus	
Yes	5 (6.3%)
No	75 (93.7%)
Vitamin D deficiency	
Yes	40 (50%)
No	40 (50%)
Osteoporosis	
Yes	41 (51.2%)
No	39 (48.8%)
Osteopenia	
Yes	29 (36.3%)
No	51 (63.7%)
Hypothyroidism	
Yes	3 (3.8%)
No	77 (96.3%)
Hypoparathyroidism	
Yes	2 (2.5%)
No	78 (97.5%)
Subclinical	
Yes	3 (3.7%)
No	77 (96.3%)
Hepatitis	
Yes	3 (3.7%)
No	77 (96.3%)
Types of hypogonadism	
Central hypogonadism	53 (96.4%)
Primary hypogonadism	2 (3.6%)

Fifty-three (66.3%) of them were female, and 27 (33.7%) were male, with a mean age of 24.86±7.66 SD (range: 14–59) years.

Fifty-five (68.75%) patients had hypogonadism, which was the most common endocrine abnormality. Forty-one (51.2%) patients were osteoporotic, while 29 (36.3%) patients had osteopenia. Osteoporosis was more common in thalassemic patients with hypogonadism TPwH and then TPwoH thalassemic patients without hypogonadism (54.54 vs. 40%).

Forty (50%) patients had vitamin D deficiency with a serum vitamin D3 (mean±SD) in TPwoH and TPwH of 18.38±15.39 ng/ml and 16.70±12.90 ng/ml, respectively.

Three patients (3.8%) had hypothyroidism, while only two (2.5%) had hypoparathyroidism.

Five patients (6.3%) had diabetes mellitus, and none had adrenal insufficiency.

Hypogonadism was more common in females than males: 42/55 (76.4%) versus 13/55 (23.6%), but the age of TPwH and TPwoH was comparable.

There was no significant difference in the compliance with the iron-chelating agents between TPwH and TPwoH (Table 2).

**Table 2:** Eugonadism versus hypogonadism.

Variable	Eugonadism *N*=25 N (%)	Hypogonadism *N*=55 (%)	*P*
Age in years (mean±SD)	25.12±10.08	24.74±6.37	0.841
Sex			
Male	14 (56)	13 (23.6)	0.004
Female	11 (44)	42 (76.4)	
Spleen			
Splenectomy	14 (56)	35 (63.6)	0.516
Normal	11 (44)	20 (64.5)	
Chelating agent			
Properly	14 (56)	32 (58.2)	0.855
Improperly	11 (44)	23 (41.8)	
Interval between two sessions of blood transfusion (mean±SD)	21.66±6.71	20.46±5.08	0.420
Diabetes mellitus			
Yes	1 (5)	4 (7.3)	0.575
No	24 (96)	51 (92.7)	
Vitamin D deficiency			
Yes	10 (40)	30 (54.5)	0.228
No	15 (60)	25 (45.5)	
Osteoporosis			
Yes	11 (44)	30 (54.5)	0.382
No	14 (56)	25 (45.5)	
Osteopenia			
Yes	9 (36)	20 (36.4)	0.975
No	16 (64)	35 (63.6)	
Hypothyroidism			
Yes	2 (8)	1 (1.8)	0.177
No	23 (92)	54 (98.2)	
Hypoparathyroidism			
Yes	1 (4)	1 (1.8)	0.562
No	24 (96)	54 (98.2)	
Subclinical hypothyroidism			
Yes	2 (8)	1 (1.8)	0.177
No	23 (92)	54 (98.2)	
Hepatitis			
Yes	0 (0)	3 (5.5)	0.234
No	25 (100)	52 (94.5)	
Ferritin (mean±SD)	1220±1785	2326±2625	0.065
Vitamin D (mean±SD)	18.38±15.39	16.70±12.90	0.657

The mean ferritin level in hypogonadism was 2326±2625 ng/ml, which was higher than its level in patients without hypogonadism, in whom it was 1220±1785 ng/ml.

Hypogonadotropic hypogonadism was more common than hypergonadotropic hypogonadism, with frequencies of 53/55 (96.36%) and 2/55 (3.63%), respectively.

There is a significant difference between TPwH and TPwoH in the level of serum testosterone in males and serum estradiol in females, respectively. Other biochemical tests are illustrated in (Table 3).

**Table 3:** Results of laboratory tests.

Tests	Eugonadism	Hypogonadism	Units
TSH	3.67±2.47	3.78±2.80	µIU/ml
FT3	6.46±2.43	5.42±1.01	ng/ml
FT4	17.14±2.88	16.64±2.86	µg/dl
Prolactin	14±6.87	9.81±4.2	ng/ml
FSH	3.46±2.06	3.96±6.39	mIU/ml
LH	5.44±4.34	6.42±12.42	mIU/ml
Calcium	9.53±0.49	9.44±9.48	mg/dl
Mg	1.94±0.07	1.97±0.42	mEq/l
Phosphorus	5.12±0.40	4.23±0.94	mg/dl
PTH	27.58±14.25	32.27±18	pg/ml
Hb	8.80±1.09	8.55±1.47	gm
WBC	16.26±10.9	16.9±11.27	10*9/l
PLT	638±261	597±302	10*9/l
Creatinine	0.52±0.13	0.51±0.12	mg/dl
ALT	28.87±27	31.66±24	u/l
AST	32±17.53	36.57±28.54	u/l
ALP	148.2±87.6	182±175	u/l
TSB	3.18±1.31	2.27±1.24	mg/dl
Albumin	4.8±0.3	4.76±0.32	g/dl
Glucose	135±114	108±29	mg/dl
E2 (Female)	84.18±42.7	39.65±26.16	pg/ml
Total testosterone (Male)	5.38±1.24	0.36±0.52	ng/ml

## Discussion

Thalassemia major is a common hereditary disease in the Kurdistan Region of Iraq, and its prevalence reached 37.3/100 000 individuals in the population in 2020^21^. This is primarily due to consanguineous marriages and a lack of preventive measures until recently.

Frequent blood transfusions result in excess iron deposition in various organs that may eventually cause dysfunction, especially in the endocrine system.

In this study, the most common manifestation of thalassemic patients referred to the endocrinology department was hypogonadism, with a frequency of 55/80 (68.75%). This is similar to what was observed in a study performed in Saudi Arabia^22^. The prevalence of hypogonadism in transfusion-dependent thalassemia varies, reaching up to 80% in some studies and being as low as 30% in others^23,24,25,26,27^. The high prevalence of hypogonadism in the present study may be related to the fact that we dealt specifically with referred patients suspected to have endocrine complications by their hematologists through history, clinical examination, and investigations.

Compliance with the chelating agents in both groups was poor and nearly the same, which can be explained by polypharmacy, the unavailability of the drugs, and a lack of awareness regarding the significance of this therapy.

There is significant heterogeneity in the epidemiology of hypogonadism in patients with thalassemia, which may be related to the differences in the clinical manifestations and characteristics^11^.

Females are more affected by hypogonadism than males, with frequencies of 42/55 (76.4%) and 13/55 (23.6%), respectively. In some studies, the same sex difference is seen^15,28^ but in others, males are more affected than females^11^.

The classification of hypogonadism depends on the level of the hypothalamic-pituitary-gonadal axis affection^29^. An inappropriately normal or low level of LH and FSH in the presence of low estradiol in females and testosterone in males indicates hypogonadotropic hypogonadism, while in hypergonadotropic hypogonadism, the level of LH and FSH is high.

In this study, hypogonadotropic hypogonadism was much more common than hypergonadotropic hypogonadism, with frequencies of 53/55 (96.36%) and 2/55 (3.63%), respectively. This is explained by the higher affinity of gonadotropic cells in the anterior pituitary for iron.

Sometimes there may be changes in both the anterior pituitary gonadotrophs and the gonads resulting from the original pathology, but it is quite difficult to differentiate these two situations, as in hypogonadotropic hypogonadism, there are changes in the gonads as well due to the loss of stimulatory effect on the gonads by LH and FSH.

Although the effect of iron on the testes and ovaries is not well understood, autopsy studies have revealed interstitial fibrosis and pigmented seminiferous tubules with an absence of Leydig cells in the testes^30^.

Many factors affect the development of hypogonadism in patients with β-thalassemia major, including chronic anemia, improper blood transfusions, improper iron chelation (either delayed or poor compliance), and other comorbidities^31,32^.

In the present study, TPwH had more comorbidities, at a frequency of 21.82% (12/55), including liver, heart, and diabetes mellitus, than TPwoH, with a frequency of 16% (4/25). This is mainly related to the same risk factors that affect the endocrine organs.

The ferritin level (mean±SD) in TPwH (2326±2625 ng/ml) was higher than TPwoH (1220±1785 ng/ml) and can be regarded as one of the risk factors for developing hypogonadism in transfusion-dependent thalassemia. Abdulzahra *et al.* and Chowdhuri *et al.* found a direct relationship between serum ferritin and the development of hypogonadism ^33,34^.

There is no significant difference in the splenectomy status in both groups, and this finding is supported by Gamberini *et al.* and Skordis *et al.* as they postulated that there is a relationship between a history of splenectomy with hypothyroidism but not hypogonadism^35,36^.

The serum level of vitamin D3 between both groups was comparable. This is compatible with the data reported by Lerchbaum E *et al.* ^37^.

The point of strength of our study is that, it exclusively dealt with the patients with thalassemia major that were suspected of having endocrinopathy, while its limitations included missing data of some patients who were excluded from the study, small size due to the limited time of the study, and measuring serum ferritin level alone as a surrogate for iron deposition.

In conclusion, hypogonadism is the most common endocrinopathy among transfusion-dependent patients with thalassemia major and is related to chronic anemia and excess iron deposition. Regular blood transfusions, proper use of chelating agents, and periodic assessment for the early detection of hypogonadism are key factors in reducing the risk of developing hypogonadism.

## Ethics approval and consent to participate

The present study was approved by Kurdistan Board for Medical Specialties Ethics Committee. Written informed consent was obtained from all participants or the parents in the case that the participants were underage.

## Patient consent for publication

Not applicable.

## Funding

No funding was received.

## Author contribution

T.O.M.: supervising the research from the title till the end; Z.G.R.: performed literature review, participated in collecting data, and reviewing grammar of the study; S.F.A.: collected the data, wrote the paper, and submitted it.

## Conflicts of interest disclosure

The authors declare that they have no competing interests.

## Research registration unique identifying number (UIN)

researchregistry9000.

## Guarantor

Shaho F. Ahmed.

## Availability of data and materials

The datasets used and analyzed during the current study are available from the corresponding author upon reasonable request.

## Provenance and Peer review

Not commissioned, externally peer reviewed.
